# Screening program for familial hyperchylomicronemia syndrome detection: Experience of a university health system

**DOI:** 10.20945/2359-3997000000601

**Published:** 2023-02-07

**Authors:** Walter Masson, Leandro Barbagelata, Milagros Fleitas, Nicole Herzkovich, Eliana Kerschner, Emiliano Rossi, Daniel Siniawski, María V. Ami, Juan P. Nogueira

**Affiliations:** 1 Hospital Italiano de Buenos Aires Servicio de Cardiología Buenos Aires Argentina Servicio de Cardiología, Hospital Italiano de Buenos Aires, Buenos Aires, Argentina; 2 Hospital Italiano de Buenos Aires Servicio de Clínica Médica Buenos Aires Argentina Servicio de Clínica Médica, Hospital Italiano de Buenos Aires, Buenos Aires, Argentina; 3 Universidad Nacional de Formosa Facultad de Ciencias de la Salud Centro de Investigación en Endocrinología, Nutrición y Metabolismo (CIENM) Formosa Argentina Centro de Investigación en Endocrinología, Nutrición y Metabolismo (CIENM), Facultad de Ciencias de la Salud, Universidad Nacional de Formosa, Formosa, Argentina

**Keywords:** Familial hyperchylomicronemia syndrome, hypertriglyceridemia, screening

## Abstract

**Objective:**

Familial chylomicronemia syndrome (FCS) is a rare autosomal recessive metabolic disorder caused by mutations related to chylomicron metabolism. The objective of this study is to show the development and results of a screening program for FCS in Argentina.

**Materials and methods:**

A cross-sectional study was performed. All patients > 18 years with a triglyceride level ≥ 1,000 mg/dL in the period from January 1, 2017 to December 31, 2021 were included. The program was developed in three stages: (1) Review of electronic records and identification of suspected laboratory cases (triglyceride level ≥ 1,000 mg/dL); (2) Identification of suspected clinical cases (all suspected laboratory cases that had no relevant secondary factors) and application of the FCS score to define probable cases (score ≥ 10); (3) Perform genetic tests in probable cases.

**Results:**

Globally, 348 suspected laboratory cases (mean age of 49.9 years, 77.3% men) were included. The median triglycerides level was 1,309 mg/dL (interquartile range 1,175-1,607 mg/dL). In total, 231 patients were categorized as suspected clinical cases. After applying the FCS score, 3% of them were classified as “very likely FCS” (probable cases). Four variants of uncertain significance have been identified. No previously reported pathogenic variants were detected.

**Conclusion:**

This study shows a screening program for the detection of FCS. Although no patient was diagnosed with FCS, we believe that more programs of these characteristics should be developed in our region, given the importance of early detection of this metabolic disorder.

## INTRODUCTION

Familial chylomicronemia syndrome (FCS) is a rare autosomal recessive metabolic disorder caused by mutations related to chylomicron metabolism (1). Thus, this congenital disease is characterized by very high fasting blood triglyceride concentrations due to chylomicronemia. In addition, extremely high triglyceride levels cause several complications in patients, the most serious being episodes of recurrent acute pancreatitis (2).

Because this condition is under-recognized, the majority of FCS patients are diagnosed after age 20, often after consulting several physicians (3). Moreover, severe hypertriglyceridemia can be observed in others metabolic conditions including type III dysbetalipoproteinemia, familial combined hyperlipidemia, familial hypertriglyceridemia and multifactorial chylomicronemia syndrome (MCS) (4).

Considering the great clinical relevance of early identification of these patients, and from a cost-effective point of view, some experts have proposed that it may be useful to have a diagnostic algorithm for FCS (5). Additionally, an expert panel proposed a diagnostic scoring system for the differential diagnosis of FCS (6). This score is based on clinical and laboratory variables, classifying patients as “very likely FCS”, “unlikely FCS” or “very unlikely FCS”.

Because FCS is a genetic condition, the optimal diagnosis strategy is genetic testing. About 80%-90% of patients with monogenic chylomicronemia have bi-allelic mutations in the lipoprotein lipase (LPL) gene (7). The remaining 10%-20% of cases are caused by monogenic variants in the genes that modulate LPL function, such as those related to apolipoprotein C2 (APOC2), apolipoprotein A5 (APOA5), glycosylphosphatidylinositol anchored high density lipoprotein binding protein 1 (GPIHBP1) and lipase maturation factor 1 (LMF1) (8).

Recently, a panel of experts proposed the following challenges related to FCS for Latin American countries: (a) to raise awareness about the disease among primary care physicians who treat these patients for the first time; (b) to promote referral to sites that can proceed with genetic tests to confirm the diagnosis; and (c) to evaluate the use of new pharmacological therapies (9).

Considering the aforementioned comments, the objective of this study was to describe a screening program for FCS with a step-wise approach in a university health system in Argentina.

## MATERIALS AND METHODS

A cross-sectional study was performed from a secondary database (electronic medical records). The sample was obtained from a private health system constituted by two university hospitals and a network of 21 associated peripheral centers distributed in Buenos Aires, Argentina.

All patients over 18 years of age who had a blood triglyceride determination ≥ 1000 mg/dL in the period from January 1, 2017 to December 31, 2021 were included.

There are several cut-off points for triglyceride level to consider “severe hypertriglyceridemia”, which especially consider the risk for pancreatitis (10,11). A triglyceride cut-off point of 1,000 mg/dL was chosen for this program because it was the definition used by the lipid clinic of our institution when the protocol was developed.

Cases were defined as follows: (a) suspected laboratory case: all cases with at least 1 fasting determination of triglycerides ≥ 1,000 mg/dL identified in the laboratory records during the evaluated period; (b) suspected clinical case: all suspected laboratory cases that had no clinically relevant secondary factors; (c) probable case: all suspected clinical cases identified with a FCS score ≥ 10. We use the score published by Moulin and cols. (6). Very likely FSC was considered when the score was ≥ 10; (d) confirmed case: all probable cases with a positive genetic test for FCS.

For this program, the following clinical situations were considered as clinically relevant hypertriglyceridemia secondary factors: (a) body mass index ≥ 35 kg/m²; (b) newly diagnosed or poorly controlled diabetes mellitus (HbA1c > 8.5%); (c) active alcoholism; (d) active hypothyroidism; (e) moderate or severe chronic kidney disease (estimated glomerular filtration rate < 60 mL/min/1.73 m²) or nephrotic syndrome; (f) pregnancy; (g) solid organ transplant with corticotherapy or immunosuppressive therapy or oral contraceptives; (h) HIV infection on long-term antiretroviral therapy.

### Program features

The program was developed in three stages: 1) Review of electronic records and identification of suspected laboratory cases; 2) Search of medical records and identification of suspected clinical cases. Then, apply the “FCS score” to define probable cases of FCS (6). In the presence of missing data, the patient was contacted by phone to complete the evaluation; 3) Perform genetic tests in probable cases, report the results and provide genetic counseling if applicable.

### Genetic analysis

Extraction and purification of genomic DNA from the submitted saliva sample was performed using the Genotek kit of Bitgenia^®^. Subsequently, the LPL, APOC2, APOA5, GPIHBP1 and LMF1 genes were analyzed. After successfully passing the quality controls, the Library was prepared, following the protocol based on capture enrichment (Library construction, SureSelect XT V6-Agilent kit). Sequencing by paired-end synthesis was carried out using the NovaSeq Sequencing System (Illumina) platform. The mapping, alignment and variant calling procedure was performed using the reference human genome GRCh37, through a protocol developed at Bitgenia^®^. Variants were identified following the Human Genome Variation Society nomenclature recommendations (12). Additionally, variants were classified according to the guidelines of the American College of Medical Genetics and Genomics (13).

### Statistical analysis

Continuous data were compared between groups using the Student's *t* test for normal distribution or the Mann-Whitney-Wilcoxon test for non-normal distribution. The analysis of categorical data was performed using the chi-square test. Continuous variables are given as mean ± standard deviation or median [25-75 interquartile range (IQR)] according to the distribution of the variables, while categorical variables are given as percentages. A value of p < 0.05 was considered statistically significant. STATA 13.0 software packages were used for statistical analysis.

## RESULTS

A total of 348 suspected laboratory cases (mean age of 49.9 ± 12.4 years, 77.3% men) were included in this program. In total, the prevalence of type 2 diabetes mellitus was 37.1% (68.2% were diagnosed before the lipid event) and 42.8% of patients were hypertensive. Average values of total cholesterol and cholesterol bound to high-density lipoproteins (HDL-C) were 305.4 ± 123.2 mg/dL and 31.5 ± 7.2 mg/dL, respectively. The median triglycerides level was 1309 mg/dL (IQR 1175–1607 mg/dL). Importantly, 5.7% of the population reported at least one previous episode of pancreatitis (seventeen patients had 1 episode, one patient had 2 episodes, and two patients had 6 episodes). The characteristics of the population can be seen in Table 1.

**Table 1:** Characteristics of the population

Continuous variables*	Total population n = 348
Age, years	49.9 (12.4)
Body mass index, kg/m^2^	30.3 (5.0)
Total cholesterol, mg/dL	305.4 (123.2)
Apolipoprotein B, mg/dL	128.4 (40.3)
HDL-C, mg/dL	31.5 (7.2)
Triglycerides, mg/dL	1,309 (1,175-1,607)
Non HDL-C, mg/dL	270.9 (105.5)
Blood glucose, mg/dL	142.6 (69.9)
Glycosylated hemoglobin (HbA1c), %**	8.2 (2.5)
**Categorical variables, %**	
Male gender	77.3
Type 2 diabetes	37.1
Hypertension	42.8
Current smoking	21.6
Pancreatitis history	5.7
Cardiovascular history	10.9
Chronic kidney disease	8.6

* Values were expressed as means (standard deviation) or median (interquartile range).

** Only in the population with diabetes.

Globally, 33.6% of the suspected laboratory cases showed a clinically relevant secondary cause (newly diagnosed or poorly controlled diabetes mellitus: 18.1%; body mass index ≥ 35 kg/m²: 10.1%; active alcoholism: 1.4%; active hypothyroidism: 1.4%; solid organ transplant: 1.1%; moderate or severe chronic kidney disease or nephrotic syndrome: 0.9%; pregnancy: 0.6%). Consequently, 231 patients were categorized as suspected clinical cases. In this subgroup of patients, the median triglycerides level was 1,309 mg/dL (IQR 1178-1591 mg/dL). In addition, 26.4%, 26.8% and 2.2% of the suspected clinical cases received fibrates, statins or omega-3 fatty acids at baseline, respectively.

The subgroup of suspected clinical cases showed no differences in terms of age or gender compared to the subgroup of subjects with clinically relevant secondary causes. Similarly, no differences were observed in the lipid profile between both groups. As expected, there were differences in the characteristics related to the definition of both groups (prevalence of diabetes or body mass index). Additionally, the suspected clinical cases showed a lower proportion of patients with hypertension, cardiovascular history or chronic kidney disease. The characteristics of both groups can be seen in Table 2.

**Table 2:** Comparative description between patients with relevant secondary causes and suspected clinical cases

Continuous variables*	Patients with relevant secondary causes (n = 117)	Suspected clinical cases (n = 231)	p
Age, years	50.5 (13.6)	49.5 (11.7)	0.481
Body mass index, kg/m^2^	32.2 (6.2)	29.3 (3.8)	<0.001
Total cholesterol, mg/dL	320.4 (145.9)	297.7 (109.3)	0.109
Apolipoprotein B, mg/dL	146.4 (67.1)	123.1 (28.7)	0.221
HDL-C, mg/dL	31.9 (7.4)	31.4 (7.1)	0.518
Triglycerides, mg/dL	1,307 (1,154-1,698)	1,309 (1,178-1,591)	0.701
Non HDL-C, mg/dL	278.1 (98.4)	267.5 (108.8)	0.416
Blood glucose, mg/dL	183.5 (81.9)	121.1 (51.1)	<0.001
Glycosylated hemoglobin (HbA1c), %**	9.5 (2.3)	7.1 (2.2)	<0.001
**Categorical variables, %**			
Male gender	81.2	75.3	0.217
Type 2 diabetes	46.2	15.6	<0.001
Hypertension	61.5	33.3	<0.001
Current smoking	26.5	19.1	0.110
Pancreatitis history	4.8	7.7	0.267
Cardiovascular history	17.1	7.8	0.009
Chronic kidney disease	15.4	5.2	0.001

* Values were expressed as means (standard deviation) or median (interquartile range).

** Only in the population with diabetes.

The median (IQR) of FCS score was 2 (-2 to 3). In total, 3% of suspected clinical cases were classified as “very likely FCS” (FCS score ≥ 10). Consequently, 7 patients were stratified as probable cases. Following the protocol, these patients were considered as candidates for genetic testing. The results of the genetic analysis showed that four variants of uncertain significance (VUS) were identified (Table 3). However, no pathogenic variant has been reported.

**Table 3:** Genetic findings

Patient	Age, sex and FCS score	Description of variant	Classification
#1	Man, 35 years old. FCS score: 11	c.878C>T [p.(Ser293Phe)] variant (heterozygosis) in the LPL gene	Uncertain significance
#2	Man, 53 years old. FCS score: 10	c.388T>C [p.(Cys130Arg)] variant (homozygosis) in the APOE gene	Non-pathogenic
#3	Woman, 34 years old. FCS score: 12	c.56C>G [p.(Ser19Trp)] variant (homozygosis) in the APOA5 gene	Non-pathogenic
#4	Man, 31 years old. FCS score: 10	c.953A>G [p.(Asn318Ser)] variant (heterozygosis) in the LPL gene	Uncertain significance
#5	Woman, 35 years old. FCS score: 10	c.953A>G [p.(Asn318Ser)]variant (heterozygosis) in the LPL gene	Uncertain significance
#6	Woman, 56 years old. FCS score: 10	c.56C>G [p.(Ser19Trp)] variant (homozygosis) in the APOA5 gene	Non-pathogenic
#7	Man, 35 years old. FCS score: 10	c.1012T>G [p.(Tyr338Asp)] variant (heterozygosis) in the LPL gene	Uncertain significance

APOA5: apolipoprotein A5; APOE: apolipoprotein E; FCS: familial chylomicronemia syndrome; LPL: lipoprotein lipase.

The alternative diagnoses established in suspected clinical cases after the end of the program were the following: (a) type III dysbetalipoproteinemia (1%); (b) familial combined hyperlipidemia (14%); (c) familial hypertriglyceridemia (23%); (d) MCS (35%); (e) secondary causes that were not considered to select the suspected clinical cases (18%): and (f) unclassified (9%).

**Figure 1:**
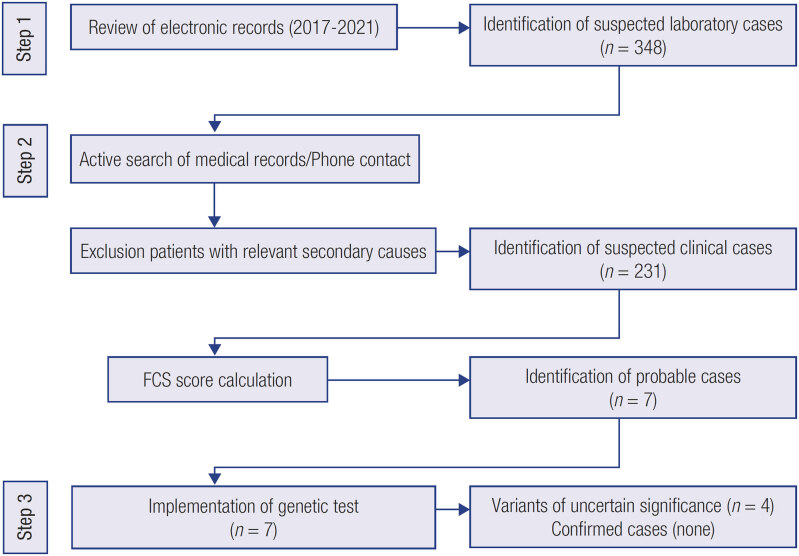
Flow diagram of the program process.

The three steps of the program can be seen in Figure 1.

## DISCUSSION

This study shows the development and results of a program specifically designed for FCS screening. Although no patient was diagnosed with this pathology, we consider that the development of programs with these characteristics are very important for our region.

This rare group of autosomal recessive disorder has an estimated population frequency of 1 per million (14). Therefore, our results were as expected if the very low prevalence of this syndrome is taken into account. In fact, most patients with severe hypertriglyceridemia have polygenic susceptibility given by the sum of common single nucleotide polymorphisms (SNPs) associated with smaller triglyceride-raising effects and not by having monogenic hypertriglyceridemia (15).

Under physiological conditions, chylomicrons are triglyceride-rich particles synthesized and secreted by enterocytes during the post absorptive state (16). These triglyceride-rich lipoproteins are cleared from the circulation by LPL located in adipose tissue and muscle capillaries where the hydrolyzed fatty acids are either stored or used as fuel. In addition, chylomicrons are converted to remnant particles by this process and are mostly taken up by the liver. Therefore, the accumulation of chylomicrons in plasma beyond the postprandial period is a pathological event secondary to the partial or complete lack of activity of LPL.

The manifestations associated with FCS are quite heterogeneous and nonspecific. A key characteristic of FCS is milky looking plasma, whereby a white chylomicron layer floats on the surface following decantation of the plasma sample (6). Additionally, high levels of circulating chylomicrons can accumulate in different locations, such as the skin (eruptive xanthomas) or the retinal blood vessels (lipemia retinalis) (17,18). Patients with FCS may also present with emotional, cognitive or psychosocial symptoms and frequently have recurrent unexplained abdominal pain. However, the most life-threatening complication of FCS is the occurrence of severe and recurrent episodes of acute pancreatitis. In addition, a recent meta-analysis of observational studies found that hypertriglyceridemia-induced acute pancreatitis is associated with a worse prognosis compared to pancreatitis of other etiology (19). In addition to the risk of recurrent pancreatitis, long-term cardiovascular risk may be increased. Although the evidence is very limited, a study published by Shah and cols. showed that there was a high rate of long-term cardiovascular events in patients with FCS despite having controlled LDL-C (<70 mg/dL) (20).

It is mandatory to exclude secondary causes when evaluating a patient with severe hypertriglyceridemia. In this program, the most common secondary causes were diabetes mellitus and obesity. The association between these secondary causes and very high triglyceride levels has been previously reported (21,22). Hypertriglyceridemia observed in patients with diabetes or obesity is often mild to moderate. Our algorithm focused the application of the Moulin score on cases without uncontrolled diabetes or extreme obesity. This is because the severity of dyslipidemia is strongly related to metabolic control, insulin resistance and central obesity. Any restriction of secondary causes probably decreases the sensitivity of screening, although at the expense of greater specificity. It is reasonable that a screening must be as sensitive as possible, but in the case of having to use genetic tests at an additional cost, it is also necessary to balance with some specificity. Primary severe hypertriglyceridemia are less frequent and include familial hypertriglyceridemia, familial combined hyperlipidemia, MCS, dysbetalipoproteinemia, and, exceptionally, FCS (3). Patients with FCS are younger and less likely to have any of the aggravating factors for hypertriglyceridemia compared to those with MCS, but are more likely to develop pancreatitis probably because of life-long, sustained chylomicronemia (23). Since the clinical characteristics between FCS and MCS tend to overlap, the score used in this study was proposed as an additional diagnostic tool. Diabetes before the lipid event was proposed as a clinical variable to distinguish between both syndromes (most frequently seen in MCS) (24). Although we did not include this clinical variable in the diagnostic algorithm, we note that the vast majority of patients with diabetes in our population were diagnosed before the finding of severe hypertriglyceridemia. Recently data suggest that in subjects at high risk of FCS a triglyceride/total cholesterol ratio or triglyceride/apolipoprotein B ratio are feasible to initially screen for type I hyperlipoproteinemia. However, the sensitivity and agreement were low for both ratios and a chylomicron/very low-density lipoprotein-triglyceride ratio was a better diagnostic criterion for FCS (25).

After analyzing a sample of 348 patients with severe hypertriglyceridemia, only 7 patients were classified as “very likely FCS” (genetic test could be done following the protocol).

Three non-pathogenic variants were reported when considering the genetic analysis. A variant in the APOA5 gene was described in two patients. APOA5 gene codes for a protein whose function is to modulate intracellular hepatic very-low-density lipoprotein (VLDL) synthesis. Likewise, it works as an activator of LPL, modulating the levels of circulating triglycerides (26). Several common single nucleotide polymorphisms (SNPs) have been described in the APOA5 gene locus. The S19W polymorphism (rs3135506; c.56C>G), includes the substitution of a cytosine for a guanine at nucleotide 56 of codon 19, which results in the change of serine residue for tryptophan (27). The association between this polymorphism and high triglyceride levels has been reported in several studies, although the causal relationship with the FCS is not yet defined (28,29).

A variant in the APOE gene was described in another patient. The APOE gene encodes the apolipoprotein E (apoE) protein, which is critical in the formation of VLDL and chylomicrons. There are three human apoE isoforms: E2, E3, and E4. E3 is the wild type allele, E4 is associated with Alzheimer disease, and E2 is associated with an increased risk for early cardiovascular disease and hyperlipoproteinemia type III (30,31). The c.388T>C [p.(Cys130Arg)] variant is known as apoE4, which is associated with increased triglyceride levels (32) However, E4/4 is not enough for cause of FCS in this patient.

On the other hand, four patients showed VUS in the LPL gene (heterozygosis) when considering the genetic analysis. One patient showed the c.878C>T variant, which produces a change in direction, generating the modified protein p.(Ser293Phe). This variant is not described in the databases consulted. Therefore, to our knowledge, it would be novel to date. Another two patients showed the c.953A>G variant, including the substitution of adenine for a guanine of exon number 6, which results in the change of asparagine residue for serine in the resulting protein [p.(Asn318Ser)]. This variant shows conflicting information in the literature, since it has been previously classified as a pathogenic, benign or VUS variant by different authors (33). Finally, one patient showed the c.1012T>G variant, including the substitution of tyrosine for a guanine of exon number 6, which results in the change of tyrosine residue for asparagine in the modified protein [p.(Tyr338Asp)]. This variant has a low population frequency (no report of homozygotes) and it has computational pathogenic prediction (based on the REVEL score) (34). However, this variant is not reported as a pathogenic variable in the current literature.

The primary therapeutic target in patients with FCS is to lower triglyceride levels and thus prevent episodes of acute pancreatitis. The mainstay of treatment is a specialized very-low-fat diet (limit fat < 15 to 20 grams per day and supplement with fat-soluble vitamins and medium-chain triglyceride oil) and fibrates (35). Unfortunately, these patients usually respond poorly to conventional therapy (4). However, several newly developed drugs might effectively treat these patients, thus improving life-long prognosis (36,37). Volanesorsen is an antisense oligonucleotide inhibitor of apoC-III designed to reduce the production of apoC-III and its efficacy was demonstrated in two recently published clinical trials (38,39). Therefore, implementing this type of program would make it possible to identify patients with FCS and evaluate new therapeutic options early.

This study presented some limitations. It was a secondary database study (electronic medical records); consequently, there could be information bias. Additionally, for this study, a triglyceride cut-off value of 1,000 mg/dL was chosen. However, taking current guidelines into account, we consider choosing a lower triglyceride value (885 mg/dL) in future programs, increasing sensitivity at the expense of lower specificity. Finally, the non-identification in our study of variants with diagnostic potential could be due to the presence of a causal variant located in a non-evaluated genetic region or to the presence of a causal variant in genes little described in the literature to date. Despite its limitations, this study represents a valuable contribution, as a large screening program of patients with severe hypertriglyceridemia.

We consider that the screening program was successful. We base on three premises: 1) the “negative” results of our study do not invalidate the usefulness of the diagnostic algorithm. When the frequency of a disease is very low, it is usual not to find patients in limited samples. Our study was carried out in a single center in Buenos Aires. It is probably necessary to evaluate many health centers concomitantly to detect a single case; 2) although we did not find pathogenic variants, we have detected cases with VUS. Many of the variants that today are classified as “pathogenic” were initially classified as VUS. Therefore, we believe that it is very important to report the genetic findings, as they could change in the future; 3) the individual impact of early detection of this pathology is extremely relevant. FCS is associated with a torpid evolution when it is not diagnosed and treated early, negatively affecting the patient, his family and the health system.

In our country, health care is highly fragmented. Replicating this type of program and incorporate screening for other primary dyslipidemias in a coordinated manner between various public and private institutions, ideally supported by scientific societies and government entities, could be a great challenge.

In conclusion, this study shows the development of a screening program designed specifically for the detection of FCS. Although no patient was diagnosed with FCS, the results are as expected considering the very low prevalence of this genetic disorder. Due to the great clinical relevance of the early detection of this metabolic disorder, we believe that it is necessary to develop and expand similar screening programs in other regions.
